# Combined detection of serum Dickkopf‐1 and its autoantibodies to diagnose esophageal squamous cell carcinoma

**DOI:** 10.1002/cam4.702

**Published:** 2016-03-14

**Authors:** Yu‐Hui Peng, Yi‐Wei Xu, Hong Guo, Li‐Sheng Huang, Hua‐Zhen Tan, Chao‐Qun Hong, Shan‐Shan Li, Li‐Yan Xu, En‐Min Li

**Affiliations:** ^1^ Department of Clinical LaboratoryThe Cancer Hospital of Shantou University Medical CollegeShantouChina; ^2^ The Key Laboratory of Molecular Biology for High Cancer Incidence Coastal Chaoshan AreaShantou University Medical CollegeShantouChina; ^3^ Department of Biochemistry and Molecular BiologyShantou University Medical CollegeShantouChina; ^4^ Department of Radiation OncologyThe Cancer Hospital of Shantou University Medical CollegeShantouChina; ^5^ Department of Oncological Research LabThe Cancer Hospital of Shantou University Medical CollegeShantouChina; ^6^ Institute of Oncologic PathologyShantou University Medical CollegeShantouChina

**Keywords:** Dickkopf‐1 (DKK‐1), DKK‐1 autoantibody, early diagnosis, esophageal squamous cell carcinoma (ESCC)

## Abstract

Esophageal squamous cell carcinoma (ESCC) can be treated effectively if diagnosed at an early stage. We evaluated whether measurement of Dickkopf‐1 (DKK‐1) in combination of DKK‐1 autoantibodies in serum may benefit early diagnosis of ESCC. Serum DKK‐1 and DKK‐1 autoantibodies were measured by enzyme‐linked immunosorbent assay in a training cohort (185 ESCC samples vs. 97 normal controls) and validated in a validation cohort (104 ESCC samples vs. 53 normal controls). Receiver operating characteristic (ROC) was applied to calculate diagnostic accuracy. Testing of DKK‐1 and DKK‐1 autoantibodies together could differentiate ESCC from normal controls (area under the ROC curve [AUC] 0.769, 95% confidence interval (CI), 0.715–0.823, 50.3% sensitivity, and 90.7% specificity in the training cohort; AUC 0.752, 95% CI, 0.675–0.829, 50.0% sensitivity, and 84.9% specificity in the validation cohort). Importantly, the diagnostic performance of the combination of DKK‐1 and DKK‐1 autoantibodies persisted in early ESCC patients (AUC 0.780, 95% CI, 0.699–0.862, 50.0% sensitivity, and 90.7% specificity in the training cohort; AUC 0.745, 95% CI, 0.626–0.865, 53.8% sensitivity, and 84.9% specificity in the validation cohort). Furthermore, the levels of serum DKK‐1 or DKK‐1 autoantibody after surgical resection were lower, respectively, compared with the corresponding preoperative samples (*P *< 0.05). Our results suggest that measurement of DKK‐1 combined with DKK‐1 autoantibodies is a potentially valuable tool for the early detection of ESCC.

## Introduction

Esophageal squamous cell carcinoma (ESCC) is one of the most common malignant diseases worldwide and especially in China, where it is the fourth leading cause of death from cancer 1, 2. The prognosis of ESCC remains poor during the last several decades, with a 5‐year overall survival rate ranging between 15% and 25% 3. Due to the lack of clinically specific symptoms of early disease and effective screening methods, most ESCC patients often present at an advanced stage when in clinics. Thus, identification of biomarkers with high sensitivity and specificity in early detection of ESCC is urgently needed and remains crucial for improving outcome.

Dickkopf‐1 (DKK‐1) is a secretory protein that inhibits the canonical Wnt signaling pathway 4. Dysregulated activation of Wnt by inhibition of DKK‐1 activity is thought to play an important role in oncogenic transformation in several cancers 5, 6. In recent years, publications have demonstrated that increased concentrations of DKK‐1 could be specifically measured in serologic samples from patients with diverse malignancies like pancreas cancer, stomach cancer, liver cancer, breast cancer, lung cancer, and so on 7, 8, 9. In fact, differential expression of serum DKK‐1 in patients with ESCC and healthy individuals were reported recently, indicating that serum DKK‐1 might be a potential biomarker for the diagnosis of ESCC 10, 11. However, those studies had one or more of the following limitations: the lack of assessments of early diagnosis value, small study size, and no independent validation.

Evidence of autoantibodies against tumor‐associated antigens (TAAs) being present in early cancer patients, or even in some individuals before developing symptomatic cancer has created opportunities for early diagnosis of cancer 12, 13, 14, 15. Considering the presence of DKK‐1 autoantibodies as a potential diagnostic biomarker in lung cancer 16, we hypothesized that the level of DKK‐1 autoantibodies is elevated in ESCC sera. On the other hand, whether measurement of combination of DKK‐1 and its autoantibody in serum could provide enhanced diagnostic efficiency for ESCC remained to be revealed. For this purpose, we examined serum DKK‐1 in combination of its autoantibody in 282 sera from 185 ESCC patients and 97 normal controls and validated the diagnostic value in an independent cohort of 104 patients and 53 normal controls.

## Materials and Methods

### Patients and samples

This study was approved by the institutional review board of the Cancer Hospital of Shantou University Medical College. Written informed consent was obtained from all participants. We consecutively recruited 289 patients who had newly diagnosed with ESCC from the Cancer Hospital of Shantou University Medical College between February 2013 and May 2014. The recruitment of ESCC patients met the following selection criteria: they were obtained before surgery from patients with biopsy proven and had received no previous anticancer treatments. Tumor stage was defined according to the seventh edition of the American Joint Committee on Cancer (AJCC) Cancer Staging Manual 17. Tumors with AJCC stage 0+I+IIA were classified as early‐stage ESCC as reported previously 14. 150 normal controls with no previous malignant disease were enrolled. On the basis of a computer‐generated allocation sequence, we randomly assigned all the participants to a training cohort (185 ESCC samples vs. 97 normal controls) or to a validation cohort (104 ESCC samples vs. 53 normal controls, Table 1). We aimed at identification of a clinically significant diagnostic biomarker from the training cohort and validated it in the independent cohort.

**Table 1:** Characteristics of the study population

Group	Training cohort (*n* = 282)				Validation cohort (*n* = 157)			
	Esophageal squamous cell carcinoma (ESCC) (*n* = 185)		Normal (*n* = 97)		ESCC (*n* = 104)		Normal (*n* = 53)	
	NO.	%	NO.	%	NO.	%	NO.	%
Age, years								
Mean	59 ± 8		50 ± 9		59 ± 9		51 ± 9	
Range	41–77		36–74		42–83		38–85	
Gender								
Male	131	71	69	71	71	68	34	64
Female	54	29	28	29	33	32	19	35
TNM stage								
0	4	2				1	1	
I	23	12				12	12	
II (IIA+IIB)	69 (25 + 44)	37				35 (13 + 22)	34	
III	85	46				53	51	
IV	4	2				3	3	
Histological grade								
High (Grade 1)	52					32	31	
Middle (Grade 2)	107					60	58	
Low (Grade 3)	18					10	10	
Unknown	8					2	3	
Depth of tumor invasion								
Tis	4	2				1	1	
T1	20	11				10	10	
T2	29	16				17	16	
T3	79	43				54	52	
T4	53	29				22	21	
Lymph node metastasis								
Positive	80	43				56	54	
Negative	105	57				48	46	
Size of tumor								
< 5 cm	85	46				53	51	
≥ 5 cm	98	52				50	58	
Unknown	2	2				1	1	
Site of tumor								
Cervical esophagus	1	1				2	2	
Upper thorax	27	15				11	11	
Middle thorax	130	70				74	71	
Lower thorax	27	15				17	16	

Peripheral blood samples were collected into anticoagulant‐free tubes before surgery and centrifuged at 1250 *g* for 5 min, and the serum was then collected and stored at −70°C until testing.

### ELISA for serum DKK‐1

Serum levels of DKK‐1 protein were measured by enzyme‐linked immunosorbent assay (ELISA) with a commercially available kit (R&D Systems, Minneapolis, MN, Catalog No. DKK100). Briefly, the concentrations of the DKK‐1 standards for creating a standard curve were 0, 31.2, 62.5, 125, 250, 500, 1000, and 2000 pg/mL. A total quantity of 100 *μ*L of Standard and sample (an eightfold dilution) were added and incubated for 2 h at room temperature, followed by the addition of 200 *μ*L of the Dkk‐1 Conjugate (a polyclonal antibody against Dkk‐1 conjugated to horseradish peroxidase [HRP]) for another 2 h at room temperature. Color development was achieved with 200 *μ*L substrate solution per well for 30 min, and sulfuric acid as stop solution was added. The optical density was read at 450 nm and referenced to 630 nm on a plate microplate reader (Multiskan MK3; Thermo Fisher Scientific). All measurements were run in duplicate. The concentrations of DKK‐1 were obtained with a standard curve, fitted for the standard value, and multiplied by the dilution factor.

### ELISA for DKK‐1 autoantibody

ELISA for serum DKK‐1 autoantibodies was performed as previously described 15. The optimal antigen coating concentration and the optimal serum dilution for the ELISA on autoantibody test were determined using a checkerboard titration in preliminary studies. Recombinant Human DKK‐1 (R&D Systems, Minneapolis, MN, Catalog No. 5439‐DK/CF) diluted to a concentration of 0.1 *μ*g/mL were dispensed in 100 *μ*L per well volumes into 96‐well microtiter plates (biohaotian, Jiangsu, China, Cat#: HT081) and incubated overnight at 4°C. A total quantity of 100 *μ*L of serum samples and quality control samples (QCS, a pooled plasma sample collected randomly from 100 patients with ESCC) were diluted 1/110 in blocking buffer, then were incubated at 37°C for 1 h, as well as appropriate control rabbit polyclonal antibodies (Immunosoft, Zhoushan, China, Cat#: IS0138) specific for capture proteins. HRP‐conjugated goat anti‐human IgG (Santa Cruz Biotechnology, Santa Cruz, CA, Cat#: sc‐2907) or anti‐rabbit IgG (Santa Cruz Biotechnology, Cat#: sc‐2054) were used as secondary antibodies at the dilution recommended by the manufacturer (1:10000). Ready‐prepared 3,3′,5,5′‐tetramethylbenzidine (TMB, InTec PRODUCTS, Xiamen, China) and hydrogen peroxide (InTec PRODUCTS) were added. After color formation, the absorbance of each well was read at 450 nm and referenced to 630 nm within 5 min by a plate microplate reader (Multiskan MK3, Thermo Fisher Scientific, Boston).

All samples were run in duplicate. The intra‐assay coefficient of variation (CV) for autoantibodies against DKK‐1 were 7.2%, and the interassay CV was 8.7%. QCSs were run to ensure quality control monitoring of the assay runs by using Levey–Jennings plots. With the purpose of minimizing an intra‐assay deviation, the ratio of the difference between duplicated sample OD values to their sum was used to assess precision of the assay. If the ratio was >10%, the test of this sample was treated as being invalid and the sample was repeated.

### Statistical analysis

All analyses were done with SPSS (version 17.0) or GraphPad Prism software. The differences in serum DKK‐1 and DKK‐1 autoantibody levels between patients and controls were assessed using a standard nonparametric Mann–Whitney's *U* test. Receiver operating characteristic (ROC) analysis was constructed to assess sensitivity, specificity, and area under the ROC curve (AUC) with the 95% confidence interval (CI). The cutoff value for positive reactivity was determined in the training set, as previously described by achieving the maximum sensitivity when the specificity was >90%, and by minimizing the distance of the cutoff value to the top‐left corner of the ROC curve. To test the diagnostic accuracy when both serum DKK‐1 and DKK‐1 autoantibody were measured, a variable predicted probability (*P*) was created on the basis of an equation obtained by binary logistic regression (all ESCC vs. all controls in the test cohort): ln[p/(1 − p)] = 0.0012 × (DKK‐1) + 4.3116 × (DKK‐1 autoantibody) − 3.6869, and the values of *P* were used as one marker and subjected to ROC analysis. The positive predictive value (PPV), negative predictive value (NPV), positive likelihood ratio (PLR), and negative likelihood ratio (NLR) were presented to improve clinical interpretation. We compared levels of serum DKK‐1 or DKK‐1 autoantibody before and after surgical resection in ESCC patients with the paired *t* test. Chi‐squared tests or Fisher's exact tests were carried out to identify correlations of individual and combined biomarker assay positivity with clinical parameters. In all tests, we considered *P* values lower than 0.05 (two‐sided) to be significant.

## Results

### DKK‐1 detection in the sera of patients with ESCC

**Figure 1:**
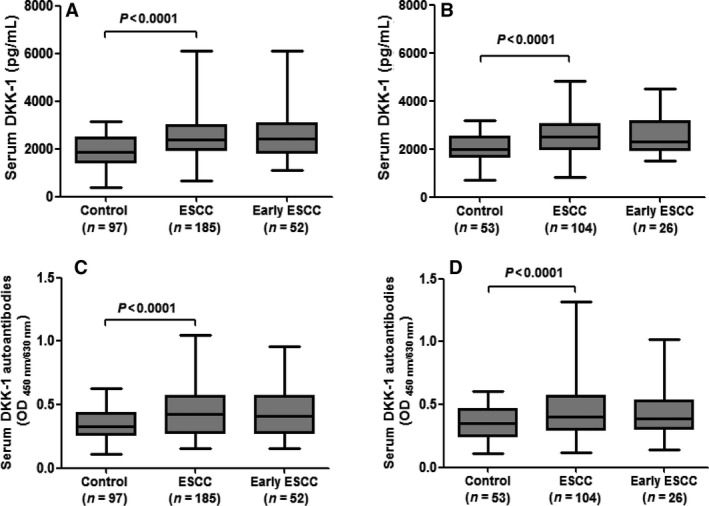
Box‐Whisker plots for levels of Dickkopf‐1 (DKK‐1) and DKK‐1 autoantibodies in serum in the training and validation cohorts. (A) DKK‐1 for training cohort. (B) DKK‐1 for validation cohort. (C) DKK‐1 autoantibodies for training cohort. (D) DKK‐1 autoantibodies for validation cohort. For each group, median levels of serum DKK‐1 or DKK‐1 autoantibodies and interquartile ranges are illustrated by box plot, and the whiskers show minimum and maximum value.

**Figure 2:**
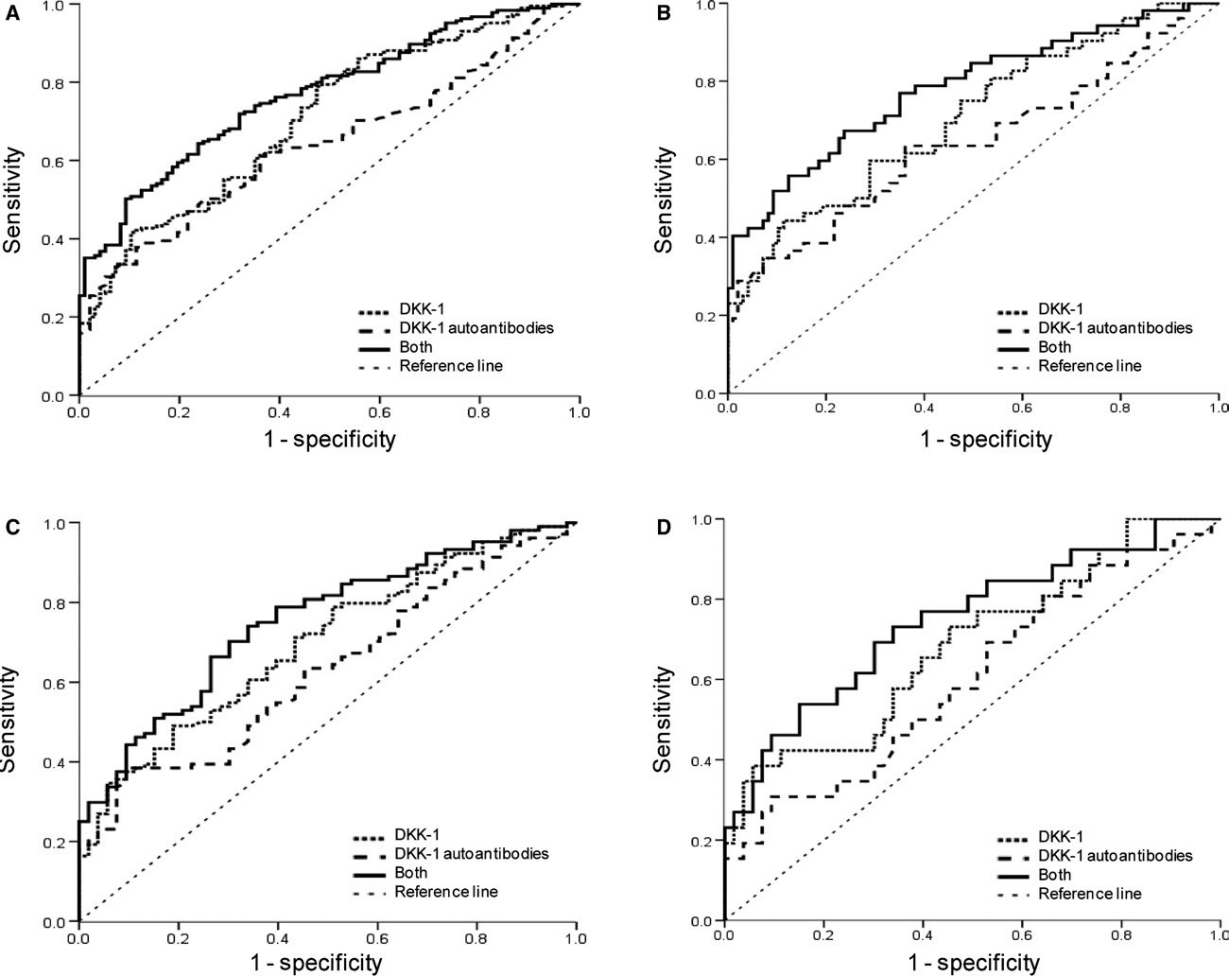
Receiver operating characteristic (ROC) curve analysis for esophageal squamous cell carcinoma (ESCC) diagnosis. (A) ROC curve for serum Dickkopf‐1(DKK‐1), DKK‐1 autoantibodies, or both for patients with ESCC versus normal controls in the training cohort. (B) ROC curve for serum DKK‐1, DKK‐1 autoantibodies or both for patients with early stage versus normal controls in the training cohort. (C) ROC curve for serum DKK‐1, DKK‐1 autoantibodies, or both for patients with ESCC versus normal controls in the validation cohort. (D) ROC curve for serum DKK‐1, DKK‐1 autoantibodies or both for patients with early stage versus normal controls in the validation cohort.

In the training cohort, DKK‐1 concentrations on ELISA were significantly higher in all ESCC patients than in controls (Fig. 1, *P* < 0.0001). We then conducted ROC curve analyses to discriminate the patients from controls (Fig. 2). With a cutoff value of 2698 pg/mL, the detection of DKK‐1 provided an AUC value of 0.709 (95% CI: 0.647–0.771), 37.3% sensitivity, and 90.7% specificity in the training cohort (Table 2). There are 52 patients with an early‐stage ESCC in the training cohort (AJCC stage 0+I+IIA). We observed similar diagnostic performance of DKK‐1 in the early‐stage ESCC patients (Fig. 2, Table 2). With use of the cutoff value for DKK‐1 from the training cohort, the results in the diagnosis of all ESCC or early‐stage ESCC were similar in the validation cohort (Figs. 1 and 2, Table 2).

**Table 2:** Results for measurement of Dickkopf‐1 (DKK‐1), DKK‐1 autoantibodies, or both in the diagnosis of esophageal squamous cell carcinoma (ESCC)

	AUC (95%CI)	Sensitivity	Specificity	PPV	NPV	PLR	NLR
Training cohort							
ESCC versus normal							
DKK‐1	0.709 (0.647–0.771)	37.3%	90.7%	88.4%	43.1%	4.011	0.691
DKK‐1 autoantibodies	0.643 (0.580–0.707)	33.5%	91.8%	88.6%	42.0%	4.085	0.724
DKK‐1 + DKK‐1 autoantibodies	0.769 (0.715–0.823)	50.3%	90.7%	91.2%	48.9%	5.409	0.548
Early‐stage ESCC versus normal							
DKK‐1	0.706 (0.618–0.794)	38.5%	90.7%	68.9%	73.3%	4.140	0.678
DKK‐1 autoantibodies	0.640 (0.541–0.739)	34.6%	91.8%	69.3%	72.4%	4.220	0.712
DKK‐1 + DKK‐1 autoantibodies	0.780 (0.699–0.862)	50.0%	90.7%	74.2%	77.2%	5.376	0.551
Validation cohort							
ESCC versus normal							
DKK‐1	0.697 (0.613–0.780)	41.3%	84.9%	84.3%	42.5%	2.735	0.691
DKK‐1 autoantibodies	0.629 (0.541–0.717)	33.7%	92.5%	89.8%	41.6%	4.493	0.717
DKK‐1 + DKK‐1 autoantibodies	0.752 (0.675–0.829)	50.0%	84.9%	86.6%	46.4%	3.311	0.589
Early‐stage ESCC versus normal							
DKK‐1	0.684 (0.557–0.812)	42.3%	84.9%	57.9%	75.0%	2.801	0.680
DKK‐1 autoantibodies	0.603 (0.468–0.739)	26.9%	92.5%	63.8%	72.1%	3.587	0.790
DKK‐1 + DKK‐1 autoantibodies	0.745 (0.626–0.865)	53.8%	84.9%	63.6%	78.9%	3.563	0.544

### Combined detection of DKK‐1 and DKK‐1 autoantibodies in patients with ESCC

We measured DKK‐1 autoantibodies in serologic samples from the training and validation cohorts and found that levels of DKK‐1 autoantibody were significantly higher in cancers than in controls (Fig. 1). The ability of DKK‐1 autoantibodies to diagnose ESCC or early‐stage ESCC was evaluated by ROC analysis, and the results suggested that DKK‐1 autoantibody may serve as a diagnostic biomarker for ESCC (cutoff value: 0.522, Fig. 2, Table 2).

We assessed whether combined detection of DKK‐1 and its autoantibodies could improve discrimination between the patients and controls. As expected, with an optimum cutoff value of 0.776, testing of both DKK‐1 and its autoantibodies effectively increased the diagnostic accuracy for ESCC compared with either test alone (AUC 0.769, 95% CI: 0.715–0.823, sensitivity 50.3%, and specificity 90.7% in the training cohort; AUC 0.752, 95% CI: 0.675–0.829, sensitivity 50.0%, and specificity 84.9% in the validation cohort; Fig. 2, Table 2). Diagnostic ability of the combination of DKK‐1 and its autoantibodies remained improved for early‐stage ESCC (Fig. 2, Table 2).

### Levels of DKK‐1 and its autoantibody in ESCC patients after surgical resection

**Figure 3:**
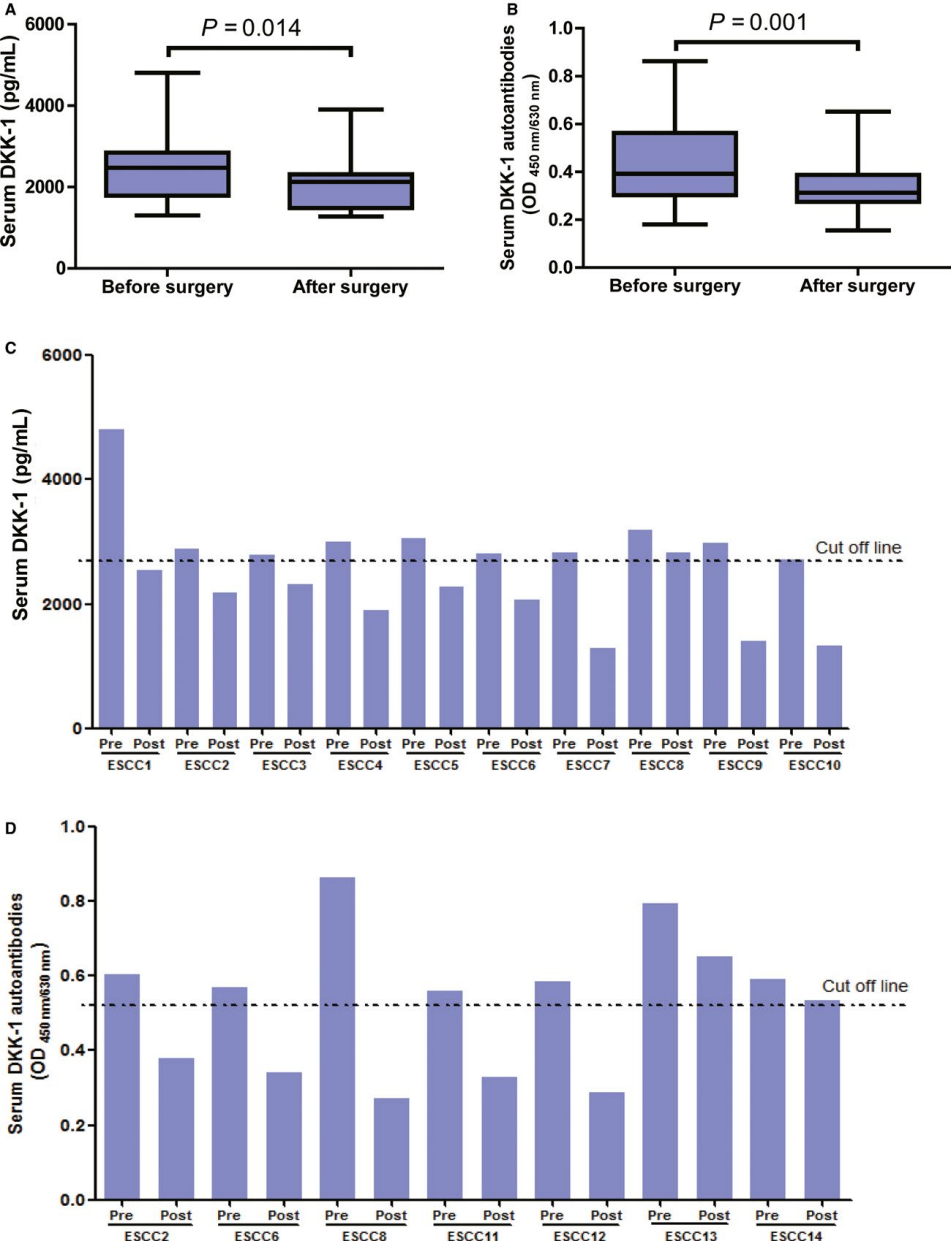
Levels of serum Dickkopf‐1 (DKK‐1) and DKK‐1 autoantibody after surgical resection of esophageal squamous cell carcinoma (ESCC). (A) Box‐Whisker plots for serum DKK‐1 levels in 21 ESCC patients before and 2 weeks after surgery. Median level and interquartile range of serum DKK‐1are illustrated by box plot, and the whiskers show minimum and maximum value. (B) Box‐Whisker plots serum levels of DKK‐1 autoantibody in 21 ESCC patients before and 2 weeks after surgery. Median level and interquartile range of DKK‐1 autoantibodies are illustrated by box plot, and the whiskers show minimum and maximum value. (C) Serum levels of DKK‐1 before and 2 weeks after surgery in ESCC patients with preoperatively positive values of serum DKK‐1. (D) Serum levels of DKK‐1 autoantibody before and 2 weeks after surgery in ESCC patients with preoperatively positive values of DKK‐1 autoantibody.

Paired preoperative and postoperative (2 weeks after the surgery) serum samples from 26 ESCC patients were investigated to monitor changes in DKK‐1 and DKK‐1 autoantibodies in the same patients. As shown in Figure 3, the levels of serum DKK‐1 or DKK‐1 autoantibody were significantly lower after surgical resection of primary tumors, compared with the corresponding preoperative samples (*P *< 0.05). Among these 26 preoperative ESCC patients, there were 10 patients and seven patients with positive values of serum DKK‐1 and DKK‐1 autoantibody, respectively. The DKK‐1 levels after tumor resection dropped below cutoff value in nine of 10 patients, and the levels of DKK‐1 autoantibody also decreased below the cutoff line in five of seven ESCC patients after treatment (Fig. 3, Table S1).

### Effect of patient characteristics on biomarker assays

In the assessment of the correlations of biomarker assays with clinical variables in ESCC patients in the training cohort and the validation cohort, DKK‐1, DKK‐1 autoantibodies or their combination showed almost no significant differences in positivity when the ESCC samples were subdivided by patient age, gender, size of tumor, site of tumor, depth of tumor invasion, histological grade, lymph node status, TNM stage or early‐stage and late‐stage groups (Tables S2, S3, and S4).

## Discussion

Currently, the diagnostic method for ESCC falls into endoscopic examination followed by histological biopsy. The invasive nature of this technique limits its application in the screening of asymptomatic populations. Thus, in the past decades, significant efforts have been made to identify robust serum or plasma markers for the early diagnosis of ESCC 18, 19, 20. However, few of these markers have been introduced into the clinical use. As such, there is clearly more work to be done for the development of novel diagnostic biomarkers for ESCC. In this study, we have demonstrated that the combined detection of serum DKK‐1 and its autoantibodies has potential diagnostic value for ESCC.

Yamabuki and colleagues 10 first reported that serum DKK‐1 should be useful as a novel diagnostic biomarker for ESCC. They noted that 51 of 81 (63.0%) ESCC patients showed positive DKK1 expression in serum. However, the sample size of their study was relative small, making it without high enough power to evaluate the diagnostic ability of DKK‐1 for this disease. Begenik et al. 11 calculated the sensitivity and specificity of 70% and 80%, respectively for serum DKK1 in the diagnosis of ESCC. However, besides the small sample size, their report was limited due to a lack of evaluation of early diagnosis value of serum DKK‐1. Furthermore, all their results have not been validated either internally or externally. Compared with above‐mentioned studies of serum DKK‐1 in the diagnosis of ESCC, our study is unique for the following respects: Firstly, we recruited 88 patients with early ESCC (52 in the training cohort and 26 in the validation cohort), which enabled us to assess the early diagnosis performance of serum DKK‐1. Furthermore, the diagnostic value of serum DKK‐1 was validated by using an independent cohort from the same medical center. This offers more strong evidence that serum DKK‐1 has the power of discrimination between ESCC or early ESCC patients and normal controls. For the difference of sensitivity and specificity between our study and others, the reasons may include the different sample size, the different proportion of patients with different tumor characteristics, and the selection of cases from different geographic origins.

In recent years, autoantibodies to TAAs have drawn increasing scientific interest owing to their promising value of clinical application in terms of the early detection of cancer 14, 21, 22. For example, EarlyCDT‐Lung, an autoantibody‐based diagnostic tool, was served as a potential complement to computed tomography for the early detection of lung cancer in routine clinical practice 21. According to Yao and colleagues in their recent report, serum levels of DKK‐1 autoantibody were higher in patients with non‐small‐cell lung cancer than in normal controls 16. Their report was the first to show that DKK‐1 as a highly immunogenic antigen could induce an autoantibody response in cancer. In this study, we also examined DKK‐1 autoantibodies in our serum samples by ELISA, and the results revealed that DKK‐1 autoantibody levels were significantly higher in ESCC patients compared to normal controls. Our finding further confirmed the presence of DKK‐1 autoantibody in cancer patients and particular relevance of autoantibodies as biomarkers for early detection.

The measurement of either DKK‐1 or its autoantibody in our study does not seem sensitive enough (i.e., high false negative rate), with sensitivity of around 30–40% in patients with ESCC and early‐stage ESCC in the training cohort (Table 2). Similar results were observed in the validation cohort (Table 2). Such a high false‐negative frequency will prevent the timely diagnosis for some ESCC patients, particularly the symptomless, early‐stage patients. Recent publications have highlighted the importance of combined analysis with multiple serum markers, which were reported to have greater sensitivity and specificity than single‐marker analysis for the detection of various cancers 9, 12, 13, 14, 15, 23. This study highlights the benefit of the combinational analysis of DKK‐1 and DKK‐1 autoantibody, which had the ability to significantly discriminate ESCC or early ESCC from normal controls, with larger AUC values compared with the markers used alone (Fig. 2, Table 2).

The postoperative levels of serum DKK‐1 in patients with hepatocellular carcinoma have been shown as a useful surveillance biomarker to evaluate the therapeutic response 9. In this study, we found decreased levels of serum DKK‐1 in most patients after tumor resection. The results support the great potential of serum DKK‐1 as a biomarker of therapeutic surveillance for ESCC. To further explore this potential role, we need to recruit a large cohort of ESCC patients who underwent surgery and undertake long‐term follow‐up of these patients. The lowered DKK‐1 autoantibody levels were also detected in most serum samples from patients who had undergone tumor resection—a finding indicating that the DKK‐1 autoantibody is attenuated on removal of the “immunogen”.

In conclusion, to the best of our best knowledge, this is the first study to analyze serum DKK‐1 combined with its autoantibodies in ESCC patients to assess their diagnostic performance. Our results suggest that serum DKK‐1 in combination of its autoantibodies could potentially aid the early detection of ESCC. Although this test is promising, further prospective and multi‐institutional studies are needed to examine the diagnostic power before it is applied clinically.

## Confict of Interest

None declared.

## Supporting information


**Table S1.** The corresponding data of DKK‐1 and its autoantibody in all the patients presented in Figure 3c and Figure 3d.
**Table S2.** Correlation between DKK‐1 and clinicopathologic characteristics of ESCC patients in both training and validation cohorts.
**Table S3.** Correlation between DKK‐1 autoantibody and clinicopathologic characteristics of ESCC patients in both training and validation cohorts.
**Table S4.** Correlation between combination of DKK‐1 and its autoantibody and clinicopathologic characteristics of ESCC patients in both training and validation cohorts.
