# Supplementary material for: Combined detection of serum Dickkopf‐1 and its autoantibodies to diagnose esophageal squamous cell carcinoma
Source: Cancer Med. 2016 Mar 14;5(7):1388–96. doi: 10.1002/cam4.702 (PMC4944864; doi:10.1002/cam4.702)
Supplement: Supplementary file 1 — Table S1. The corresponding data of DKK‐1 and its autoantibody in all the patients presented in Figure 3c and Figure 3d. Table S2. Correlation between DKK‐1 and clinicopathologic characteristics of ESCC patients in both training and validation cohorts. Table S3. Correlation between DKK‐1 autoantibody and clinicopathologic characteristics of ESCC patients in both training and validation cohorts. Table S4. Correlation between combination of DKK‐1 and its autoantibody and clinicopathologic characteristics of ESCC patients in both training and validation cohorts. [file CAM4-5-1388-s001.docx]

| **Supplementary Table S1. The corresponding data of DKK-1 and its autoantibody in all the patients presented in Figure 3c and Figure 3d** | | | | |
| --- | --- | --- | --- | --- |
| Patient No. | Serum DKK-1 (pg/ml) | | DKK-1 autoantibody (OD _450nm/630nm_) | |
|  | Pre-operation | Post-operation | Pre-operation | Post-operation |
| ESCC1 | 4808.1 | 2538.3 |  |  |
| ESCC2 | 2891.8 | 2188.2 | 0.603 | 0.379 |
| ESCC3 | 2786.4 | 2312.7 |  |  |
| ESCC4 | 2997.3 | 1892.6 |  |  |
| ESCC5 | 3054.8 | 2281.6 |  |  |
| ESCC6 | 2805.6 | 2063.8 | 0.570 | 0.342 |
| ESCC7 | 2834.4 | 1278.1 |  |  |
| ESCC8 | 3191.8 | 2818.4 | 0.863 | 0.271 |
| ESCC9 | 2973.9 | 1410.3 |  |  |
| ESCC10 | 2709.4 | 1332.5 |  |  |
| ESCC11 |  |  | 0.560 | 0.329 |
| ESCC12 |  |  | 0.585 | 0.287 |
| ESCC13 |  |  | 0.795 | 0.652 |
| ESCC14 |  |  | 0.592 | 0.535 |

| **Supplementary Table S2. Correlation between DKK-1 and clinicopathologic characteristics of ESCC patients in both training and validation cohorts** | | | | | | | | | |  |
| --- | --- | --- | --- | --- | --- | --- | --- | --- | --- | --- |
|  | Training cohort | | | |  | Validation cohort | | | | |
|  | n | Positive (%) | *X* | *P* |  | n | Positive (%) | *X* | *P* | |
| Patient age |  |  |  |  |  |  |  |  |  | |
| ≤ 60 | 106 | 35 (33.0) | 1.943 | 0.163 |  | 59 | 24 (40.7) | 0.025 | 0.874 | |
| > 60 | 79 | 34 (43.0) |  |  |  | 45 | 19 (42.2) |  |  |  |
| Patient gender |  |  |  |  |  |  |  |  |  | |
| Male | 131 | 41 (31.3) | 6.908 | 0.009 |  | 71 | 28 (39.4) | 0.336 | 0.562 | |
| Female | 54 | 28 (51.8) |  |  |  | 33 | 15 (45.5) |  |  |  |
| Size of tumor |  |  |  |  |  |  |  |  |  | |
| <5 cm | 85 | 34 (40.0) | 0.785 | 0.376 |  | 53 | 20 (37.7) | 0.418 | 0.518 | |
| ≥5 cm | 98 | 33 (33.7) |  |  |  | 50 | 22 (44.0) |  |  |  |
| Site of tumor |  |  |  |  |  |  |  |  |  | |
| Upper thorax | 27 | 8 (29.6) | 0.749 | 0.688 |  | 11 | 4 (36.4) | 0.121 | 0.941 | |
| Middle thorax | 130 | 50 (38.5) |  |  |  | 74 | 31 (41.9) |  |  |  |
| Lower thorax | 27 | 10 (37.0) |  |  |  | 17 | 7 (41.2) |  |  |  |
| Depth of tumor invasion |  |  |  |  |  |  |  |  |  | |
| T1+T2 | 49 | 16 (32.7) | 0.692 | 0.405 |  | 27 | 13 (48.1) | 0.616 | 0.432 | |
| T3+T4 | 132 | 52 (39.4) |  |  |  | 76 | 30 (39.5) |  |  | |
| Histological grade |  |  |  |  |  |  |  |  |  | |
| High (Grade 1) | 52 | 18 (34.6) | 5.451 | 0.066 |  | 32 | 13 (40.6) | 0.489 | 0.783 | |
| Middle (Grade 2) | 107 | 35 (32.7) |  |  |  | 60 | 25 (41.7) |  |  | |
| Low (Grade 3) | 18 | 8 (44.4) |  |  |  | 10 | 3 (30.0) |  |  |  |
| Lymph node metastasis |  |  |  |  |  |  |  |  |  | |
| Positive | 80 | 29 (36.3) | 0.066 | 0.797 |  | 56 | 25 (44.6) | 0.544 | 0.461 | |
| Negative | 105 | 40 (38.1) |  |  |  | 48 | 18 (37.5) |  |  | |
| TNM stage |  |  |  |  |  |  |  |  |  | |
| I | 23 | 7 (30.4) | 0.806 | 0.668 |  | 12 | 4 (33.3) | 0.432 | 0.806 | |
| II | 69 | 28 (40.6) |  |  |  | 35 | 14 (40.0) |  |  | |
| III | 85 | 31 (36.5) |  |  |  | 53 | 23 (43.4) |  |  | |
| Early-stage VS. late-stage |  |  |  |  |  |  |  |  |  | |
| Early(0+I+IIA) | 52 | 20 (38.5) | 0.042 | 0.838 |  | 26 | 11 (42.3) | 0.013 | 0.908 | |
| Late(IIB+IIIA+IIIB+IIIC) | 133 | 49 (36.8) |  |  |  | 78 | 32 (41.0) |  |  | |

| **Supplementary Table S3. Correlation between DKK-1 autoantibody and clinicopathologic characteristics of ESCC patients in both training and validation cohorts** | | | | | | | | | | |  |
| --- | --- | --- | --- | --- | --- | --- | --- | --- | --- | --- | --- |
|  | Training cohort | | | |  |  | Validation cohort | | | | |
|  | n | Positive (%) | *X* | *P* | |  | n | Positive (%) | *X* | *P* | |
| Patient age |  |  |  |  | |  |  |  |  |  | |
| ≤ 60 | 106 | 32 (30.2) | 1.232 | 0.267 | |  | 59 | 16 (27.1) | 2.608 | 0.106 | |
| > 60 | 79 | 30 (38.0) |  |  |  |  | 45 | 19 (42.2) |  |  |  |
| Patient gender |  |  |  |  | |  |  |  |  |  | |
| Male | 131 | 43 (32.8) | 0.096 | 0.757 | |  | 71 | 24 (33.8) | 0.002 | 0.962 | |
| Female | 54 | 19 (35.2) |  |  |  |  | 33 | 11 (33.3) |  |  |  |
| Size of tumor |  |  |  |  | |  |  |  |  |  | |
| <5 cm | 85 | 31 (36.5) | 0.476 | 0.490 | |  | 53 | 17 (32.1) | 0.043 | 0.836 | |
| ≥5 cm | 98 | 31 (31.6) |  |  |  |  | 50 | 17 (34.0) |  |  |  |
| Site of tumor |  |  |  |  | |  |  |  |  |  | |
| Upper thorax | 27 | 7 (25.9) | 2.232 | 0.328 | |  | 11 | 6 (54.5) | 2.884 | 0.236 | |
| Middle thorax | 130 | 42 (32.3) |  |  |  |  | 74 | 25 (33.8) |  |  |  |
| Lower thorax | 27 | 12 (44.4) |  |  |  |  | 17 | 4 (23.5) |  |  |  |
| Depth of tumor invasion |  |  |  |  | |  |  |  |  |  | |
| T1+T2 | 49 | 14 (28.6) | 0.791 | 0.374 | |  | 27 | 6 (22.2) | 1.926 | 0.165 | |
| T3+T4 | 132 | 47 (35.6) |  |  | |  | 76 | 28 (36.8) |  |  | |
| Histological grade |  |  |  |  | |  |  |  |  |  | |
| High (Grade 1) | 52 | 19 (36.5) | 1.039 | 0.595 | |  | 32 | 10 (31.3) | 1.472 | 0.479 | |
| Middle (Grade 2) | 107 | 32 (29.9) |  |  | |  | 60 | 23 (38.3) |  |  | |
| Low (Grade 3) | 18 | 7 (38.9) |  |  |  |  | 10 | 2 (20.0) |  |  |  |
| Lymph node metastasis |  |  |  |  | |  |  |  |  |  | |
| Positive | 80 | 26 (32.5) | 0.065 | 0.799 | |  | 56 | 21 (37.5) | 0.804 | 0.370 | |
| Negative | 105 | 36 (34.3) |  |  | |  | 48 | 14 (29.2) |  |  | |
| TNM stage |  |  |  |  | |  |  |  |  |  | |
| I | 23 | 7 (30.4) | 0.724 | 0.696 | |  | 12 | 2 (16.7) | 3.347 | 0.188 | |
| II | 69 | 21 (30.4) |  |  | |  | 35 | 9 (25.7) |  |  | |
| III | 85 | 31 (36.5) |  |  | |  | 53 | 21 (39.6) |  |  | |
| Early-stage VS. late-stage |  |  |  |  | |  |  |  |  |  | |
| Early(0+I+IIA) | 52 | 18 (34.6) | 0.039 | 0.843 | |  | 26 | 7 (26.9) | 0.703 | 0.402 | |
| Late(IIB+IIIA+IIIB+IIIC) | 133 | 44 (33.1) |  |  | |  | 78 | 28 (37.3) |  |  | |
|  | | | | | | | | | | |  |

| **Supplementary Table S4. Correlation between combination of DKK-1 and its autoantibody and clinicopathologic characteristics of ESCC patients in both training and validation cohorts** | | | | | | | | | | |
| --- | --- | --- | --- | --- | --- | --- | --- | --- | --- | --- |
|  | Training cohort | | | |  |  | Validation cohort | | | |
|  | n | Positive (%) | *X* | *P* | |  | n | Positive (%) | *X* | *P* |
| Age |  |  |  |  | |  |  |  |  |  |
| ≤ 60 | 106 | 45 (42.5) | 1.220 | 0.269 | |  | 59 | 27 (45.8) | 0.585 | 0.444 |
| > 60 | 79 | 40 (50.6) |  |  |  |  | 45 | 24 (53.3) |  |  |
| Gender |  |  |  |  | |  |  |  |  |  |
| Male | 131 | 57 (43.5) | 1.597 | 0.206 | |  | 71 | 36 (50.7) | 0.248 | 0.618 |
| Female | 54 | 29 (53.7) |  |  |  |  | 33 | 15 (45.5) |  |  |
| Size of tumor |  |  |  |  | |  |  |  |  |  |
| <5 cm | 85 | 41 (48.2) | 0.531 | 0.466 | |  | 53 | 22 (41.5) | 2.163 | 0.141 |
| ≥5 cm | 98 | 42 (42.9) |  |  |  |  | 50 | 28 (56.0) |  |  |
| Site of tumor |  |  |  |  | |  |  |  |  |  |
| Upper thorax | 27 | 11 (40.7) | 1.386 | 0.500 | |  | 11 | 7 (63.6) | 3.717 | 0.156 |
| Middle thorax | 130 | 58 (44.6) |  |  |  |  | 74 | 38 (51.4) |  |  |
| Lower thorax | 27 | 15 (55.6) |  |  |  |  | 17 | 5 (29.4) |  |  |
| Depth of tumor invasion |  |  |  |  | |  |  |  |  |  |
| T1+T2 | 49 | 21 (42.9) | 0.341 | 0.559 | |  | 27 | 15 (55.6) | 0.534 | 0.465 |
| T3+T4 | 132 | 63 (47.7) |  |  | |  | 76 | 36 (47.4) |  |  |
| Histological grade |  |  |  |  | |  |  |  |  |  |
| High (Grade 1) | 52 | 26 (50.0) | 3.127 | 0.209 | |  | 32 | 16 (50.0) | 0.844 | 0.656 |
| Middle (Grade 2) | 107 | 41 (38.3) |  |  | |  | 60 | 27 (45.0) |  |  |
| Low (Grade 3) | 18 | 10 (55.6) |  |  |  |  | 10 | 6 (60.0) |  |  |
| Lymph node metastasis |  |  |  |  | |  |  |  |  |  |
| Positive | 80 | 38 (47.5) | 0.137 | 0.711 | |  | 56 | 27 (48.2) | 0.033 | 0.856 |
| Negative | 105 | 47 (44.8) |  |  | |  | 48 | 24 (50.0) |  |  |
| TNM stage |  |  |  |  | |  |  |  |  |  |
| I | 23 | 12 (52.2) | 0.562 | 0.755 | |  | 12 | 3 (25.0) | 3.022 | 0.221 |
| II | 69 | 32 (46.4) |  |  | |  | 35 | 14 (40.0) |  |  |
| III | 85 | 37 (43.5) |  |  | |  | 53 | 27 (50.9) |  |  |
| Early-stage VS. late-stage |  |  |  |  | |  |  |  |  |  |
| Early(0+I+IIA) | 52 | 26 (50.0) | 0.479 | 0.489 | |  | 26 | 14 (53.8) | 0.321 | 0.571 |
| Late(IIB+IIIA+IIIB+IIIC) | 133 | 59 (44.4) |  |  | |  | 78 | 37 (47.4) |  |  |
|  | | | | | | | | | | |
